# Synthesis of Some Eco-Friendly Materials for Gold Recovery

**DOI:** 10.3390/polym16172512

**Published:** 2024-09-04

**Authors:** Theodora Babău, Mihaela Ciopec, Narcis Duteanu, Adina Negrea, Petru Negrea, Nicoleta Sorina Nemeş, Bogdan Pascu, Maria Mihăilescu, Catalin Ianasi

**Affiliations:** 1 Faculty of Industrial Chemistry and Environmental Engineering, Politehnica University of Timişoara, Victoriei Square, No. 2, 300006 Timisoara, Romania; theodora.babau@student.upt.ro (T.B.);; 2 Renewable Energy Research Institute–ICER, Politehnica University of Timisoara, Gavril Musicescu Street, No. 138, 300774 Timisoara, Romaniaioan.pascu@upt.ro (B.P.);; 3 “Coriolan Drăgulescu” Institute of Chemistry, Bv. Mihai Viteazul, No. 24, 300223 Timisoara, Romania

**Keywords:** gold, adsorption, recovery, amino acids, cellulose, chitosan, diatomea

## Abstract

The aim of this study was to develop new materials with adsorbent properties that can be used for the adsorption recovery of Au(III) from aqueous solutions. To achieve this result, it is necessary to obtain inexpensive adsorbent materials in a granular form. Concomitantly, these materials must have a high adsorption capacity and selectivity. Other desired properties of these materials include a higher physical resistance, insolubility in water, and materials that can be regenerated or reused. Among the methods applied for the separation, purification, and preconcentration of platinum-group metal ions, adsorption is recognised as one of the most promising methods because of its simplicity, high efficiency, and wide availability. The studies were carried out using three supports: cellulose (CE), chitosan (Chi), and diatomea earth (Diat). These supports were functionalised by impregnation with extractants, using the ultrasound method. The extractants are environmentally friendly and relatively cheap amino acids, which contain in their structure pendant groups with nitrogen and sulphur heteroatoms (aspartic acid—Asp, l-glutamic acid—Glu, valine—Val, DL-cysteine—Cys, or serine—Ser). After preliminary testing from 75 synthesised materials, CE-Cys was chosen for the further recovery of Au(III) ions from aqueous solutions. To highlight the morphology and the functionalisation of the material, we physicochemically characterised the obtained material. Therefore, the analysis of the specific surface and porosity showed that the CE-Cys material has a specific surface of 4.6 m^2^/g, with a porosity of about 3 nm. The FT-IR analysis showed the presence, at a wavelength of 3340 cm^−1^, of the specific NH bond vibration for cysteine. At the same time, pHpZc was determined to be 2.8. The kinetic, thermodynamic, and equilibrium studies showed that the pseudo-second-order kinetic model best describes the adsorption process of Au(III) ions on the CE-Cys material. A maximum adsorption capacity of 12.18 mg per gram of the adsorbent material was achieved. It was established that the CE-Cys material can be reused five times with a good recovery degree.

## 1. Introduction

Waste produced from different industrial activities represents a significant threat to human life because it can cause significant environmental problems. Due to the actual socioeconomical content, gold containing waste sharply increases dramatically, causing problems from an economic and environmental point of view. In this context, special attention must be paid to the development of new technologies capable of recovering gold from different secondary products [1,2]. Gold and gold combinations represent a raw material in different processes in some industries, such as the chemical industry, jewellery industry, and electronic industry, but also in medicine. Due to its physicochemical properties, gold and its combinations are used as catalysts [3,4]. Currently, approximately 75% of pure gold is produced using gold ore as a raw material [5]. In the actual context of environmental protection and circular economy, a huge amount of electronic waste must be recycled to minimize our carbon footprint. It is well-known that modern electronic devices contain different valuable metals, so such wastes must be used as secondary sources for all these metals. There are a number of well-established processes used for gold recovery from secondary sources, such as liquid–liquid extraction, coprecipitation, membrane separation, ion exchange, solvent extraction, or adsorption. All of these processes are characterized by a relatively good efficiency, but some of them are responsible for some environmental pollution [6,7]. Among the processes mentioned above, it was proven that adsorption represents the best option due to its higher efficiency, which is correlated with a friendly relationship with the environment, flexibility, and cost [7,8].

An increasing number of adsorbents have been designed for gold recovery from aqueous solutions, but most of them are relatively expensive, having a low selectivity and significant impact on the environment. Examples of such materials with adsorbent properties used for gold recovery are adsorbent polymers, graphene materials, metal organic frameworks (MOFs), nanotubes, nanocomposites [9,10,11,12,13], functionalized polymers, nanosilica [14], chitosan with a modified structure, cellulose modified with hydrazono-imidazoline [15,16,17,18], thiourea-modified Cu_2_O, pyridine-modified chitosan, and geopolymers [19]. The preparation of such materials involves higher production costs and laborious methods. Compared to all these synthetic adsorbents, natural biosorbents such as chitosan or cellulose exhibit good tolerance in a large pH range, higher selectivity, adaptability, and a good regeneration ability [20]. However, these natural sorbents present a major disadvantage, which is represented by a relatively low efficiency in their native form; therefore, their functionalization with different active groups is needed [21,22].

An important issue is represented by the development of new eco-friendly adsorbents with a high efficiency and good recycling features. Growing interest for such adsorbent materials was translated into the research field by using natural polymers as raw materials for the further development of eco-friendly adsorbents. A green adsorbent material must be prepared using renewable or recycled materials as feedstock; these materials are transformed by processes that have a minimal chemical or energy footprint, presenting a higher stability so the materials do not induce a supplemental contamination and can be easily reused as many times as possible [23].

Liu et al. have synthesized the materials used as raw materials for the synthesis of green adsorbent materials. They are as follows: agricultural waste (husks, straw, crop leftovers, coconut shell, wood chips, etc.), animal wastes (manure, hair, bones, etc.), low-cost mineral resources, industrial wastes (red mud, fly ask, blast furnace sludge, etc.), and municipal solid wastes [23].

For example, for humic acid elimination, Wang, S. et al. developed green nano-composite adsorbents; these adsorbents were prepared using Fe3O4-chitosan modified with phenyl-alanine [24]. In another work, Mullaimalar et al. prepared a geopolymer adsorbent by using copper slag and rice husk ash as raw materials. This new material was tested as an effective adsorbent for methylene blue removal [25]. Kim, W.T. et al. prepared a new eco-friendly adsorbent material by producing a boehmite layer (AlOOH) on top of aluminium sheets. This new adsorbent material was used with good results for fluoride removal [26]. In a similar approach, Fekry, M. et al. prepared an eco-friendly adsorbent material with chitosan/magnetite particles modified with abietic or boswellic acids for the removal of waste oil from a water surface [27].

Technological advancement leads to higher quantities of industrial waste waters with a metallic ion content. Strict environmental regulations, correlated with resource depletion, transform these waters as a possible resource. Starting from this, Kim, J. et al. produced and tested an eco-friendly Cu(II) adsorbent material based on PVA/PAA nanofibers containing thiol-modified silica particles [28]. Similar, Bai, Y. et al. prepared an eco-friendly adsorbent material for heavy metal ion recovery based on a silsesquioxane core and poly 92-dimethyl aminoethyl methacrylate [29]. As was demonstrated, chitosan and starch are polyvalent polysaccharides having different applications. Alvarado, N. et al. [30] identified that these polymers are highly demanded for adsorbent material preparation.

Zhang, G. et al. prepared and tested an efficient adsorbent for uranium recovery from water by modifying the MIL 101 metal organic framework with glycine, histidine, or cysteine [31]. Hu, G. et al. prepared an adsorbent for gold recovery by using metal organic frameworks in which they incorporate amino acids [32]. Similarly, Peng, L. et al. prepared a new adsorbent material for Pd(II) recovery by the functionalization of cellulose microspheres with different amino acids (arginine, histidine, methionine, and cysteine) [33]. A different approach was represented by the usage of immobilized lanmodulin (a natural protein from methylorubrum extorquens) for the separation of Sc, Y, La, and Lu [34].

To develop new materials with adsorbent properties, they must meet the following requirements: (i) they must be low-cost, (ii) they must have the possibility of being obtained in granular form, (iii) they must have a high adsorption capacity and selectivity, (iv) they must be able to present a higher physical resistance (not to disintegrate in water), and (v) they must be able to be regenerated for reuse in multiple adsorption cycles [35]. The adsorptive properties of a material can be improved by chemically modifying it or by functionalizing it with different extractants containing pendant groups with different heteroatoms (nitrogen, sulphur, phosphorus, or oxygen atoms), which act as active adsorption centres. Such materials are usually in liquid form or can be brought into a liquid state by dissolving them in a proper solvent (it is advisable that the extractant presents minimal solubility in the chosen solvent). Organic and inorganic materials can be used as support for hte functionalization process. Thus, macroporous polymers or silica matrices with a rigid three-dimensional structure can be used as support materials, being suitable for the incorporation of large amounts of the desired extractant. In order to obtain high-performance adsorbent materials, it is important that the used support material has a good specific surface area and a high mechanical resistance. Before, functionalization is necessary in order to prepare support materials by washing, drying, or swelling procedures. It is desirable that, following these preparatory processes, the properties of the used support material do not change [36,37]. The main advantages of using porous supports are represented by the higher adsorption capacity (due to the fast kinetics) and the possibility of easy regeneration of the spent adsorbent materials [38,39,40,41,42,43,44].

Ultrasonication represents a new method used to keep the support and extractant in contact through an agitation movement created by ultrasonic waves (>20 kHz). Ultrasonic waves propagated in the liquid medium result in alternating high-pressure cycles (compression) with low-pressure one (rarefaction). During the rarefaction cycle, voids appear in the liquid medium, which then collapse violently, producing the so-called cavitation [45]. Similarly, during the compression cycle, very high local temperatures are created. During ultrasonication, due to the cavitation effect, the contact surface between the support and neighbourhood liquid increases. The cavitation process occurs at several points simultaneously, generating high temperatures and pressures, which lead to physicochemical changes in the support and extractant [46,47]. The cavitation process can be classified as an acoustic, optical, or hydrodynamic one. Acoustic cavitation is responsible for the intense increase in temperature, unlike hydrodynamic cavitation. At the same time, cavitation can also be classified as a transient and/or stable one [48]. Transient cavitation occurs as a result of the fact that the cavitation free space is filled with vapours generated at ultrasonic intensities higher than 10 W cm^−2^, a phenomenon that leads to a change in the size of the formed bubble, also changing their lifetime to a few seconds [49]. Energy dissipated during cavitation cycle can affect the used support due to the generation of different hot spots [50]. The increase in surface area observed during ultrasonication could lead to the splitting of hydrogen bonds from the support structure, thus improving the interaction with extractant molecules, improving, in this way, the functionalization process. This method presents the advantage of achieving a more efficient contact between the solid support and liquid extractant, thus achieving a higher functionalization degree. Another important advantage is represented by the shorter contact time compared to the dry method. The main disadvantage of this method is that the material requires subsequent drying, in order to be used further in adsorption processes [48,50,51,52,53,54,55].

The aim of this study is to synthesize, through functionalization, through impregnation, some new, environmentally friendly adsorbent materials, starting from cellulose, chitosan, and diatomaceous as supports, and different amino acids (aspartic acid, l-glutamic acid, valine, DL-cysteine, or serine) as extractants, in order to recover gold ions by adsorption from aqueous solutions. Thus, of the total number of synthesized materials, only one proved to be the most effective for Au(III) ion recovery by adsorption from aqueous solutions. The selected material was characterized by physicochemical methods in order to highlight the functionalization of the used support, by impregnation. At the same time, the surface change was also evaluated by determining the pHpZc of the synthesized material. Kinetic, thermodynamic, and equilibrium studies were carried out for this new prepared material.

## 2. Materials and Methods

### 2.1. Adsorbent Synthesis

The purpose of present study was to obtain new materials with adsorbent properties through functionalization by impregnation. Thus, 75 new materials were synthesized. As material support, three different biopolymers were used: cellulose—CE (Avicel PH-101, Sigma Aldrich, Saint Louis, Missouri, US), chitosan—Chi (Carl Roth, Karlsuhe, Germany), and diatomaceous—Diat (diatomaceous earth, FutuNatura, Kranj, Slovenia). As extractants, five different amino acids were used, containing NH_2_ and COOH groups in their structure. These amino acids are aspartic acid (Asp), L-glutamic acid (Glu), valine (Val), DL-cysteine (Cys), and serine (Ser) (all these acids being purchased from Sigma Aldrich).

Ultrasonication functionalization was carried out in an ultrasound bath (Sonorex Super 10 P Bandelin, Bandelin electronic GmbH and Co KG, Berlin, Germany), the contact time between solid support and extractant being 10 min, the ultrasound frequency being 50 Hz, and the temperature being 298 K. Furthermore, obtained suspension was filtered using a vacuum pump and dried for 24 h at 323 K.

**Figure 1:**
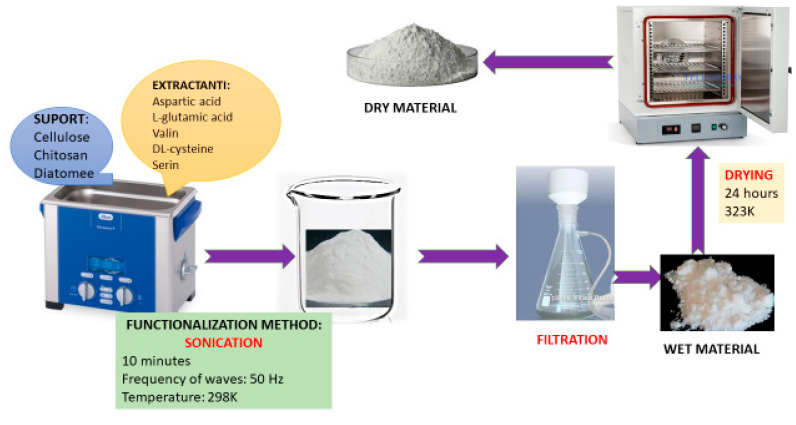
Schematic representation of adsorbent material preparation through ultrasonication.

To obtain the desired adsorbent materials, 1 g of support was weighed, over which different amounts of extractants (between 0.05 and 0.25 g) were added, which was previously dissolved in 25 mL of acidified distilled water, to obtain different support:extractant mass ratio (1:0.05, 1:0.10, 1:0.15, 1:0.20, and 1:0.25, respectively). A schematic representation of the material preparation is presented in Figure 1.

### 2.2. Adsorbent Testing for Au(III) Recovery

After synthesis of the adsorbent material, all 75 obtained materials were tested as adsorbent materials for Au(III) ion recovery from aqueous solutions. The adsorption capacity of each new prepared adsorbent material was evaluated by determining the residual amount of A(III) ions in the residual solution obtained after adsorption, by atomic adsorption spectrometry (AAS) using the Varian 120 FS AAS spectrophotometer (Varian Inc., Palo Alto, CA, USA).

Therefore, approximately 0.1 g of new prepared adsorbent material has been weighed, over which 25 mL of the solution with an initial content of 20 mg Au(III) ions per liter was added. In this preliminary stage, all adsorptions were carried out under the following conditions: pH range between 2 and 4, temperature of 298 K, and a contact time of 60 min.

In addition, the adsorption capacity is evaluated using the following relation:

$$q=\frac{(Ci-Crez)\cdot V}{m}\;\;\left[mgg^{-1}\right]$$

where: C_i_—Au(III) ions initial concentration, mg L^−1^;

C_rez_—Au(III) residual concentration, mg L^−1^;

V—solution volume, L;

m—adsorbent mass, g.

Following the results obtained after the preliminary adsorption study, we were able to determine that the best affinity for Au(III) was presented by the material designed as CE-Cys. This material was chosen for all the following studies.

### 2.3. Selected Adsorbent Characterization, CE-Cys

After preliminary studies, a new prepared adsorbent material designated as CE-Cys was selected as the best candidate for any subsequent adsorption studies. In first stage, new prepared adsorbent material has been characterized by scanning electron microscopy (SEM) and energy-dispersive X-ray spectroscopy (EDX) by using a Quanta FEG 250 electron microscope (FEI, Hilsboro, OR, USA). After that, CE-Cys material was characterized by Fourier-transformed infrared spectroscopy (FT-IR) by using a Jasco FT/IR—4200 spectrophotometer (SpectraLab Shimadzu, Kyoto, Japan).

At the same time, the pHpZc of the CE-Cys adsorbent material was determined by bringing the studied system to equilibrium [56,57]. For this study, a quantity of 0.1 g of CE-Cys adsorbent material was brought in contact with 25 mL of 0.01 M KCl solution, and kept in contact for 60 min at 200 rotation per minute at 298 K. The pH of the solution was adjusted in the range of 1–14 using NaOH solutions with a concentration between 0.05 and 2 N, or HNO_3_ solutions with concentrations between 0.05 and 2 N. After experiments, the samples were filtered, and, later, the pH of the obtained solution was determined by using a Crison MultiMeter MM41 pH-meter. To evaluate the pHpZc value, the final pH value (pH_f_) was plotted against the initial value (pH_i_).

### 2.4. Au(III) Recovery Mechanism by Adsorption onto CE-Cys Material

#### 2.4.1. Kinetic Studies

To highlight the kinetics of Au(III) recovery process by adsorption, but also for a better understanding of the kinetic mechanism that governs Au(III) ion adsorption on CE-Cys adsorbent material, obtained experimental data were modelled using two different kinetic models: the pseudo-first-order kinetic model (Lagergren kinetic model) and the pseudo-second-order kinetic model (Ho–McKay kinetic model).

#### 2.4.2. The Influence of the S:L Ratio on the Efficiency of the Adsorption Process

An important parameter, which has an important influence on Au(III) adsorption process, is represented by ratio between adsorbent material used and the volume of the solution containing Au(III) ions used during the adsorption experiments. By varying this ratio, we established the optimal amount of adsorbent material necessary for carrying out the adsorption process of Au(III) ions with a maximum yields. This was achieved by determining the adsorption capacity of the prepared material at a pH ~2 using a gold solution with an initial concentration of 20 mg L^−1^ and a contact time of 60 min, at 200 rpm, and with a temperature of 298 K. To achieve this objective, different quantities of adsorbent material—CE-Cys (0.025, 0.05, 0.75, 0.10, 0.15, 0.20, 0.30, 0.40, 0.50, and 0.60 g) were weighed and further were contacted with 25 mL of solution containing 20 mg Au(III) per L. When the experiment was finished, all samples were filtered and the gold residual concentration was determined by AAS.

#### 2.4.3. pH Influence

It is well-known that the pH represents the control parameter for all adsorptive processes and can be influenced not only by the ionic form of gold, but also by the nature of the functional groups founded on the surface of the adsorbent materials. Present studies were carried out by adjusting the pH in the range 2 to 12. In this case, the pH values were adjusted by using HNO_3_ or NaOH solutions with concentrations ranging between 0.05 and 2 M. Gold solution with an initial concentration of 20 mg L^−1^ was obtained by dilution from a stock solution with a concentration of 1 g L^−1^. Each sample was prepared by accurately weighing 0.1 g of adsorbent material (CE-Cys), over which 25 mL of Au(III) solution with desired pH was added. Obtained samples were stirred in a water bath for 60 min at 298 K. After that, samples were filtered and the residual concentration of Au(III) ions in the filtrate was determined by using AAS. 

#### 2.4.4. Contact Time and Temperature

To determine the influence of the contact time and temperature on the adsorption capacity of CE-Cys adsorbent material, for each sample, 0.1 g of material was accurately weighed. After that, 25 mL of gold solution with an initial concentration of 20 mg L^−1^ with pH 2 was added. Adsorbent material was kept in contact with gold solution for different times (15, 30, 45, 60, 90, and 120 min), but also at different temperatures (298, 308, 318, and 328 K). All samples were stirred at a rotation speed of 200 rpm. Samples were then filtered, and the residual concentration of gold was determined as previously.

Kinetic models are used to identify the type of the adsorption mechanism for the studied system and to identify potential stages, and to evaluate the speed of the studied processes, including mass transport processes and possible chemical reactions [58]. Most frequently used are pseudo-first-order kinetic model (Lagergren model) [59] and pseudo-second-order kinetic model (Ho and McKay model) [60,61,62]. The kinetic equations used to express these models are as follows:

Lagergren kinetic model is expressed by [59]:

$$\ln\left(q_{e}-q_{t}\right)=\ln q_{e}-k_{1}t$$

where: *q_e_*—equilibrium adsorption capacity, mg g^−1^;

*q_t_*—adsorption capacity at a specific time *t*, mg g^−1^;

*k*_1_—pseudo-first-order speed constant, min^−1^;

*t*—contact time, min.

Ho and McKay kinetic model is expressed by [60,61,62]:

$$\frac{t}{q_{t}}=\frac{1}{k_{2}q_{e}^{2}}+\frac{1}{q_{e}}$$

where: *q_e_*—equilibrium adsorption capacity, mg g^−1^;

*q_t_*—adsorption capacity at a specific time *t*, mg g^−1^;

*k*_2_—pseudo-second-order speed constant, g mg^−1^ min^−1^;

*t*—contact time, min.

From the equation associated with the graphical representation of the linear dependence between ln(*q_e_* − *q_t_*) as a time function, the values of the pseudo-first-order rate constant and the calculated adsorption capacity are evaluated. Similarly, from the equation of the line obtained from the graphical representation of the linear dependence *t*/*q_t_* as a time function, the pseudo-second-order rate constant and the calculated adsorption capacity are evaluated. The model that describes most accurately the studied adsorption process is established based on calculated kinetic parameters and based on the values of the correlation coefficients (R^2^).

#### 2.4.5. Thermodynamic Studies

Activation energy can be evaluated by using the Arrhenius equation [63] and the rate constant calculated from the pseudo-second-order kinetic model (k_2_), which is specific for the adsorption of Au(III) ions on the CE-Cys adsorbent material. Activation energy is calculated from the equation associated with the linear dependence between ln *k*_2_ = f(1/T). In the next stage, in order to elucidate how Au(III) ions are adsorbed on the CE-Cys adsorbent material, the value of free Gibbs energy is calculated by using Gibbs–Helmholtz equation [63,64]. Standard enthalpy and standard entropy variations associated with the studied adsorption process are evaluated using van ’t Hoff equation [63]. The equilibrium constant of the gold adsorption process is represented by the ratio between the adsorption capacity at equilibrium and the equilibrium concentration.

#### 2.4.6. Equilibrium Studies

In order to carry out the equilibrium studies, it is important to evaluate the way that the initial concentration of gold ions influence the adsorption capacity of the studied adsorbent material. Therefore, we established the value of the maximum initial concentration of gold ions which lead to a maximum adsorption capacity. These studies were carried out by varying the Au(III) ion initial concentration in the range of 5 to 180 mg L^−1^, for a contact time of 60 min, at 298 K and pH ~2 (optimal conditions established during previous studies).

On the basis of the data obtained from the adsorption experiments, adsorption isotherms can provide information regarding the maximum adsorption capacity of materials with adsorbent properties, but also can indicate a possible mechanism of the adsorptive process. In present study, the classical adsorption isotherms, Langmuir, Freundlich, and Sips isotherms, were used [65,66,67,68]. The langmuir isotherm is based on the assumption that the adsorption is taking place in a monomolecular layer. Similar, the Freundlich isotherm was developed to describe the adsorption on heterogeneous adsorbent surfaces. The Sips isotherm represent a combination of Langmuir and Freundlich isotherms [65,66,67,68]. All the information regarding these isotherms (assumptions, non-linear/linear equations, and constants) are presented in [63,69]. Specific parameters for each isotherm are obtained from the slopes of linear representations and from the ordinate at origin.

#### 2.4.7. CE-Cys Material Reuse Studies

In order to evaluate the efficiency of the adsorbent material in successive adsorption/desorption processes and to establish the maximum number of adsorption/desorption cycles, a dynamic adsorption process was used. In this context, a fritted glass column with a diameter of 2 cm and a height of 6 cm was loaded with 10 g of adsorbent material. Over this column, a solution with the maximum admitted concentration (established during the experiments—160 mg L^−1^) with a constant flow rate of 0.1 L h^−1^ was passed. To evaluate the adsorption column breakthrough, sequences of samples of 10 mL each were taken. The residual concentration of Au(III) ions was determined from these sequences. 

In addition, several adsorption/desorption cycles were performed until the adsorbent material was exhausted, thus establishing the maximum number of adsorption/desorption cycles. Desorption experiments were carried out by using a 5% HNO_3_ solution.

## 3. Results and Discussion

### 3.1. Testing as an Adsorbent for Gold Recovery

**Figure 2:**
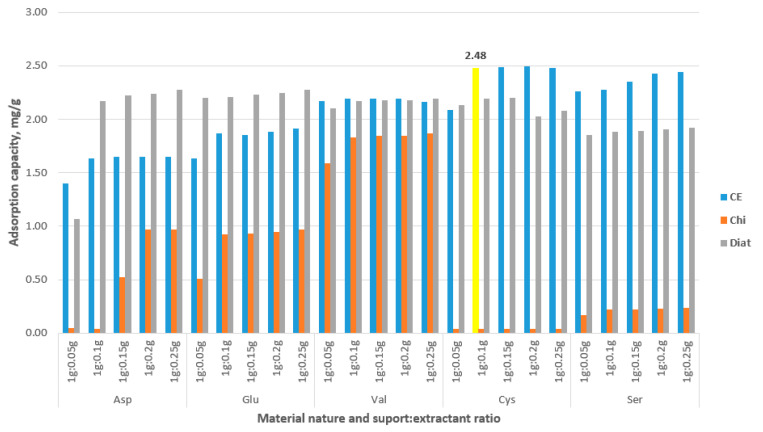
Adsorption capacities obtained for the prepared adsorbent materials.

Figure 2 presents the adsorption capacities obtained for the new synthesized adsorbent materials, tested for the recovery of Au(III) ions from aqueous solutions.

Analyzing the data presented in Figure 2, we can observe that all the extractants used for support functionalization present some affinity for Au(III) ions from aqueous solution. In addition, we can observe that cellulose, but also diatomaceous, can be considered to be the supports that “support” with very good results the extractants used for A(III) recovery by adsorption from aqueous solutions. From the obtained experimental data, we can observe that the maximum adsorption capacity of 2.48 mg Au(III) per g of adsorbent material was obtained for the adsorbent material obtained by the functionalization of cellulose with cysteine (material further designed as CE-Cys). At the same time, it can be stated that the adsorption capacity increases with the increase in the ratio of support:extractant. From the experiments carried out, we can conclude that the optimum ratio is 1:0.1, since, at higher ratios, the adsorption capacity presents no significant increase. For further studies. the adsorbent material obtained by the functionalization of cellulose with cysteine was selected.

### 3.2. Characterization of New Synthesized CE-Cys Adsorbent Material

**Figure 3:**
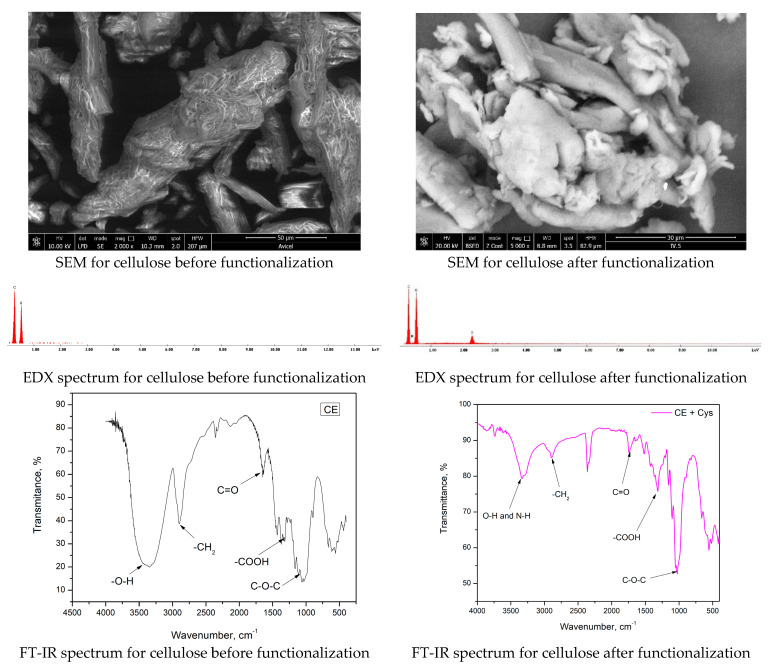
Physico-chemical characterization of CE-Cys adsorbent material.

The new prepared adsorbent material was characterised by physico-chemical methods in order to highlight the presence of active groups (-NH_2_, -SH, and –COOH) on the cellulose surface, confirming, in this way, his functionalization. Therefore, in Figure 3, we present the SEM micrographs, EDX, and FT-IR spectra recorded for raw and functionalized cellulose.

A change in the cellulose surface morphology subsequent functionalization can be observed by analyzing the data presented in the SEM micrograph recorded before and after functionalization. At the same time, the recorded EDX spectrum reveals the presence of nitrogen and sulphur atoms, which confirms the functionalization of cellulose with DL-cysteine.

From the FT-IR spectra, we can observe the presence of a peak located at wave number 3340 cm^−1^ which can be associated with the stretching vibration of O-H bonds, bonds specific to the cellulose structure [70], but also with the vibration of N-H bonds specific to cysteine [71]. For the peak located at 2900 cm^−1^, it is associated with the vibration of the group –CH_2_ [72], a group specific to the cellulose molecule. The vibration located at 1730 cm^−1^ is associated with the vibration specific to the C=O bonds [71,73], which are specific to both molecules (cellulose and cysteine). At wave number 1311 cm^−1^ is located the signal specific to the –COOH group, which is presented in both molecules, and at 1026 cm^−1^ is located the vibration specific for C-O-C bonds [74] from cellulose molecules.

### 3.3. Determination of Material pH_pZc_

**Figure 4:**
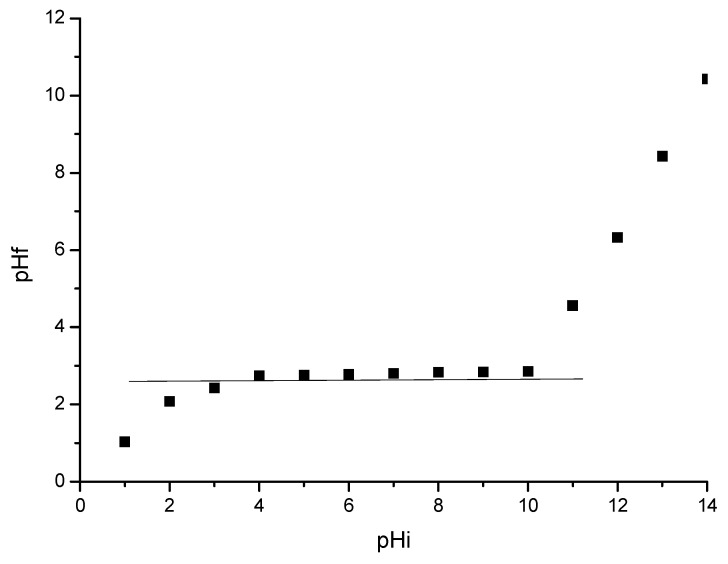
pH_pZc_ for CE-Cys material.

In Figure 4, we present the dependence between pHf and pHi used to determine the value of pHpZc for the prepared CE-Cys adsorbent material.

From the data presented in Figure 4, we can observe that, for each value of the initial pH between 4 and 10, the used adsorbent material present the buffering capacity, meaning that the final pH has a constant value of 2.8 corresponding to the surface zero charge potential.

### 3.4. Au(III) Recovery Mechanism by Adsorption onto CE-Cys Material

#### 3.4.1. The Influence of the S:L Ratio on the Efficiency of the Adsorption Process

**Figure 5:**
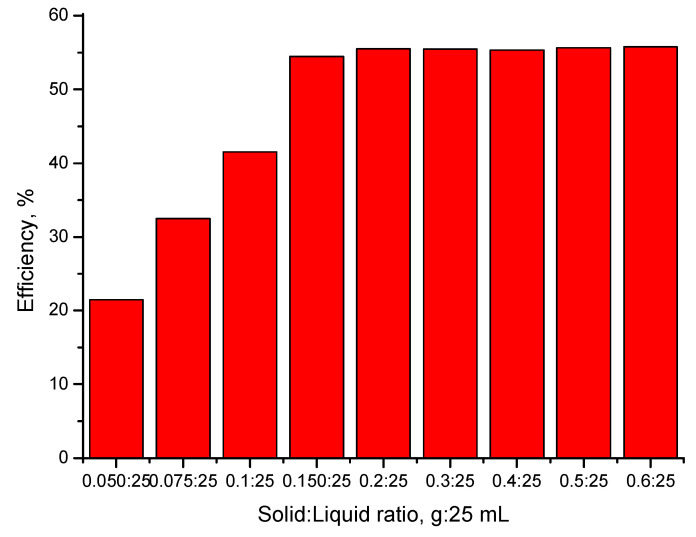
S:L ratio influence.

The experimental data regarding the influence of the S:L ratio on the efficiency of the adsorption process are presented in Figure 5.

From the data presented in Figure 5, we can observe that. by increasing the S:L ratio, the efficiency of the adsorptive process increases until a maximum efficiency is reached. After that, any further increases in the ratio lead to no increase in the gold recovery efficiency. The obtained experimental data demonstrate that the maximum efficiency was obtained for an S:L equal with 0.1 g:25 mL.

#### 3.4.2. pH Influence

**Figure 6:**
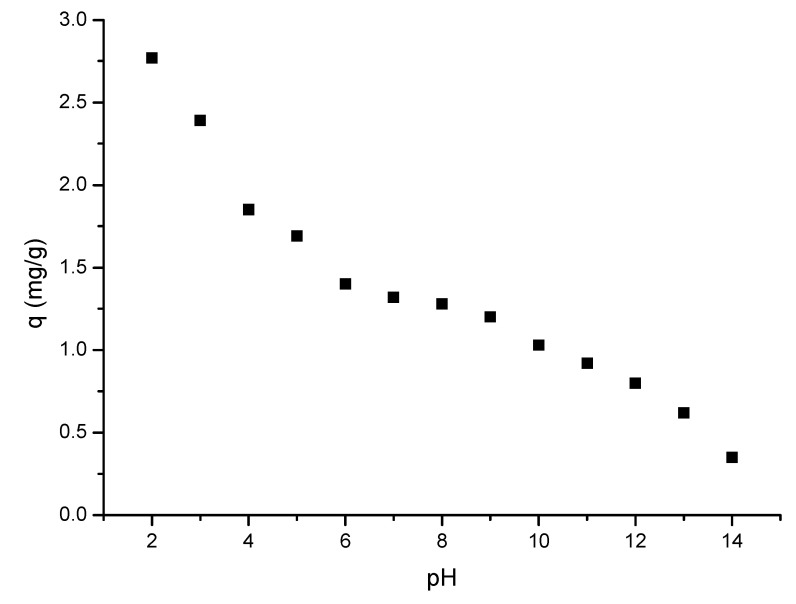
pH influence on adsorption capacity.

In Figure 6 are presented the experimental data obtained from studies regarding the influence of the pH value on gold recovery adsorption.

The experimental data presented in Figure 6 indicate a decrease in the adsorption capacity concomitant with the pH increase. From the obtained experimental data, we can observe that the adsorptive process takes place with good results at a pH lower than 4. Moreover, it can be observed that, at a pH higher than 4, the adsorption capacity exhibits a sharp decrease. At a low pH (when the pH value was controlled with HCl), a good adsorption capacity can be explained by the presence of enough chloride ions to favour the formation of Au(III) chloro-anionic species that are adsorbed by protonated groups of the L-glutamic amino acid [75]. The adsorption of chloro-anionic species can be due to the electrostatic interaction with protonated aminic groups presented into the studied adsorbent material, increasing, in this way, the number of free adsorptive active centres able to bind the metal ion complex [76]. In the presence of AuCl_4_^−^, the interactions between the gold complex and the active centres can be as follows:

(R-NH_3_ ^+^)Cl^−^ + AuCl_4_^−^ ‹-› (R-NH_3_ ^+^)AuCl_4_^−^ + Cl^−^

The obtained results indicate that the Au(III) ion adsorption is taking place with good the efficiency in the pH range between 1 and 4, when the predominant species that is adsorbed is AuCl_4_^−^ [69,77,78,79,80,81,82]. 

#### 3.4.3. Contact Time and Temperature Influence

**Figure 7:**
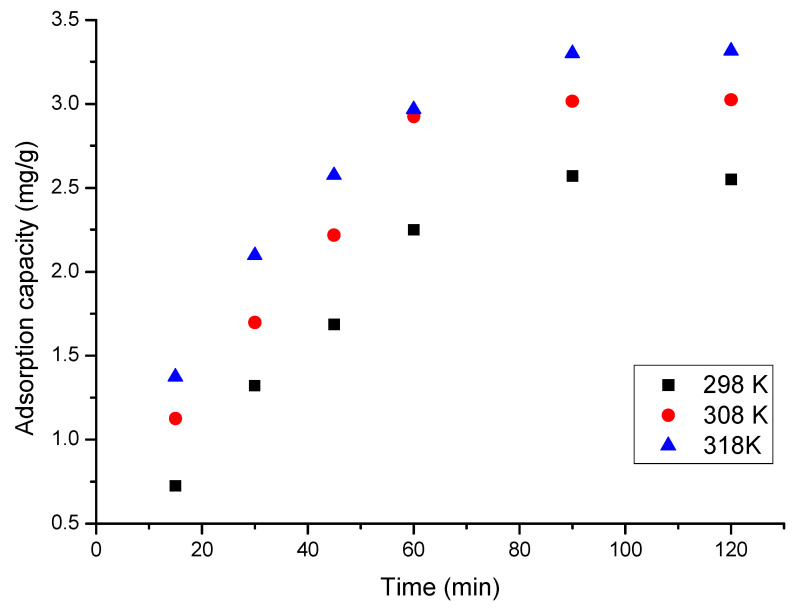
Contact time and temperature influence.

The Au(III) ion adsorption capacity on CE-Cys depends on the contact time and temperature at which the adsorptive process takes place. At the same time, we studied the influence of the contact time at different temperatures in order to establish the kinetics of the adsorption process, but also to determine the associated kinetics parameters. The obtained experimental data are depicted in Figure 7.

Taking a look at the data presented in Figure 7, it can be observed that, with the increase in contact time, an increase in the adsorption capacity of the CE-Cys adsorptive material occurs until a constant value is reached. A similar behaviour is observed if the temperature increases. From the data presented in Figure 7, it can be observed that, after 90 min, the value of the adsorption capacity remains relatively constant, being between 2.6 and 3.6 mg Au(III) ions per g of adsorbent material.

#### 3.4.4. Kinetic Studies

To analyze the kinetics of the Au(III) adsorption process and also to obtain a better understanding of the kinetic mechanism that also governs the Au(III) ion adsorption process on CE-Cys, the obtained experimental data were modelled using two different kinetic models: the pseudo-first-order (Lagergren) model and pseudo-second-order (Ho and McKay) model.

**Figure 8:**
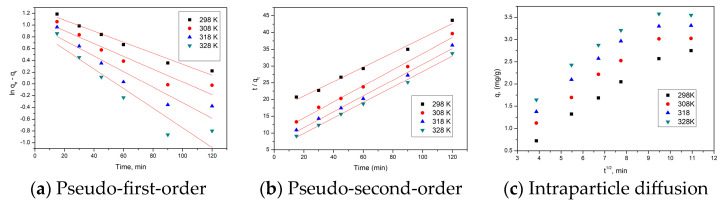
Kinetic studies for Au(III) ion adsorption on CE-Cys adsorbent.

Thus, the linearized form presented in Figure 8a,b are obtained by the graphical representation of the linearized form of these models for the experimental data obtained at the four working temperatures.

To distinguish whether the film diffusion or intraparticle one represents the rate-determining step, the obtained experimental data were modelled by using the Weber and Morris model (the obtained data are presented in Figure 8c). It is observed that Au(III) ion adsorption takes place in several stages, because the lines obtained by the graphical representation of the dependence *q_t_* = f(*t*^1/2^) at different temperatures do not pass through the origin (C = 0). On the basis of this observation, we can state that both diffusions (the film and intraparticle one) influence the studied adsorptive process.

In Table 1 are presented the kinetic parameters obtained by modelling the experimental data with selected models.

A value of the correlation coefficient (R^2^) closer to unity means that the model for which the value was obtained better describes the studied adsorption process. Another factor that influences the choice of the kinetic model that describes the adsorption process is the experimentally obtained adsorption capacity (q_e,exp_), which must be close to the one calculated by using the kinetic model (q_e,calc_). Analysing the data presented in Table 1, it is observed that the correlation factor is closest to 1 when the experimental data were modelled by using the pseudo-second-order kinetic model. Moreover, we can observe that q_e,calc_ is close to q_e,exp_. As a result of these results, we can affirm that the pseudo-second-order kinetic model best describes the Au(III) ion adsorption process on CE-Cys.

In addition, from the data presented in Table 1, it can be observed that, with the increase in temperature, the value of K_diff_ also increases. The diffusion constants for step 1 are higher than the diffusion constants for step 2, which means that step 2 is the rate-determining one.

#### 3.4.5. Thermodynamic Studies

**Figure 9:**
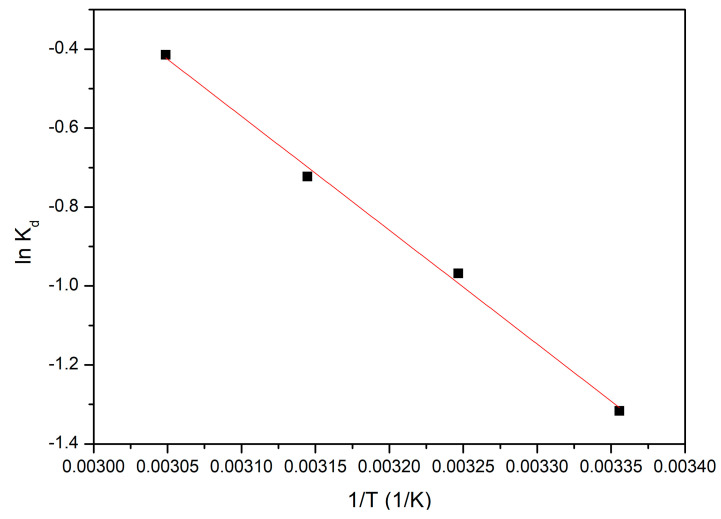
Thermodynamic studies.

In order to establish the information related to the energy changes associated with the studied adsorption process, thermodynamic studies were carried out in the temperature range of 298 to 328 K. Based on the data obtained from the thermodynamic studies, it can be concluded whether the studied adsorption process is spontaneous or not. In this context, the variations of enthalpy (ΔH^0^), free Gibbs energy (ΔG^0^), and free enthalpy (ΔS^0^) were determined. Entropy and enthalpy variations were determined from the linear dependence of ln K_d_ = f(1/T) (Figure 9). Later, based on these values, the value of the free Gibbs energy was calculated by using the van ’t Hoff equation.

The associated thermodynamic parameters are presented in Table 2.

The positive value of the standard enthalpy variation demonstrates that the energy required for the adsorption process is represented by the energy used to bring Au(III) ions in contact with the surface of the adsorbent material. When this material is dispersed in the solution containing Au(III) ions, the hydrogen atoms from water molecules interact intensively with the groups from the extractant molecules that contain oxygen atoms, forming, in this way, a relatively high number of hydrogen bonds. Such interactions block the active centres for the adsorption process by the presence of water molecules, thus limiting the access of Au(III) ions at the active centres. At the same time, the affinity shown by the CE-Cys material toward Au(III) ions can be explained by taking into account the appearance of electrostatic or complexing interactions, usually through endothermic processes. This observation is consistent with the favourable effect of temperature on the adsorption process. The calculated value of the free Gibbs energy is negative, indicating that the studied adsorptive process is a spontaneous and natural one. The positive value of the variation in the adsorption entropy suggested that the speed of the adsorption process increases at the adsorbent/solution interface. At the same time, with the temperature increase, there is an increase in the degree of disorder of the system. This change can be attributed to the changes that occur on the surface of the adsorbent material. On the basis of all these observations, we can conclude that the Au(III) adsorption on the surface material is an endothermic and spontaneous process.

#### 3.4.6. Activation Energy

The activation energy represents the minimum of the kinetic energy that the reactant must possess in order to undergo chemical transformations. With regard to the adsorption processes, the activation energy is defined as the minimum amount of energy that the adsorbate particles must possess, so their adsorption on the adsorbent becomes possible. To understand the mechanism by which the adsorption process is driven, it is important to evaluate how the intermolecular forces influence the development of the studied process [83]. When the studied molecules possess a small amount of kinetic energy or collisions occurs at improper angles, the desired process does not take place, with collisions being ineffective. If the molecules have an energy greater than the minimum energy barrier (E_a_—activation energy) and if the collisions occur under a favourable spatial orientation, the adsorption process takes place.

**Figure 10:**
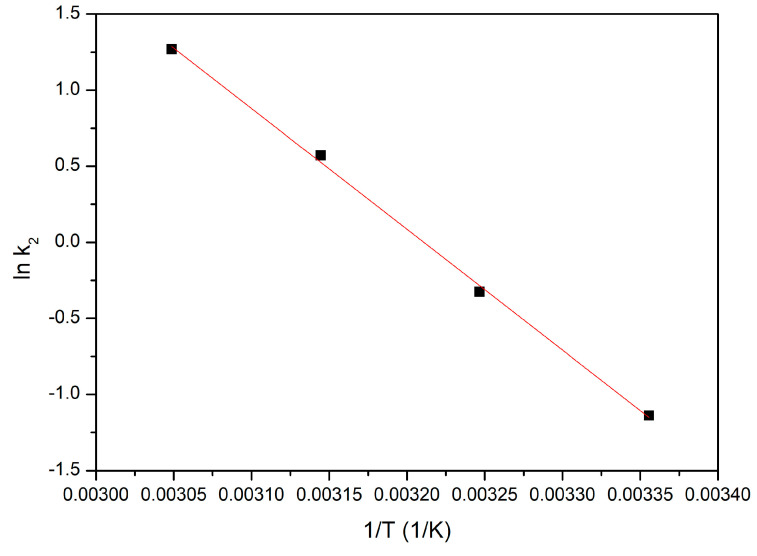
Dependence lnK_2_ vs 1/T used for determination of E_a_.

Thus, in order to evaluate the value of E_a_ associated with the studied adsorption process, the linear dependency ln K_2_ = f(1/T) was represented (Figure 10). In this graphical representation, the value of K_2_ is the value of the rate constant obtained when the experimental data were modelled by using the pseudo-second-order kinetic model.

Based on the data presented in Figure 10, it was determined that the activation energy has a value of 3.13 kJ mol^−1^, and the correlation coefficient was 0.9887. Taking into account the value of the activation energy, we can conclude that the studied adsorption process is a physical one [84].

#### 3.4.7. Equilibrium Studies

**Figure 11:**
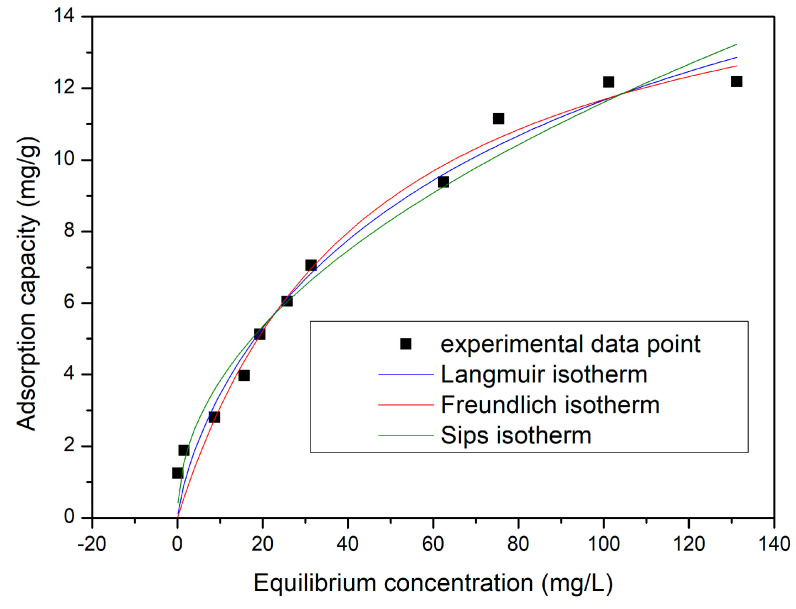
Adsorption isotherms obtained for Au(III) adsorption on CE-Cys.

For a better understanding of the studied adsorption process, it is necessary to identify the mechanism that drives the adsorption, by describing the way in which the solution interacts with the adsorbent material. This can be achieved by modelling the experimental data with the equilibrium isotherms, which present the relationship between the amount of adsorbed substance per gramme of adsorbent, at equilibrium (q_e_), and the concentration of Au(III) ions remaining in the aqueous phase (Ce). To describe the Au(III) adsorption process obtained, the experimental data were modelled by using the Langmuir, Freundlich, and Sips adsorption isotherms (Figure 11).

By modelling the obtained experimental data, the parameters specific for each adsorption isotherm were obtained (Table 3).

From data from the existing literature [85], it can be stated that most of the Au(III) ion adsorption process on the CE-Cys adsorbent is a multilayer process, and the material surface is a heterogeneous one. At the same time, the adsorption mechanism is controlled by a chemisorption process because of the strong chelation between the metal ions and HO- groups or the free electron pairs of the pendant groups that have N and/or S atoms, founded on the material surface. Evaluating the value of the heterogeneity factor, n_s_, which usually has a value lower than 2, allows us to state that there is or is not the possibility and probability that the adsorption process takes place through the migration of Au(III) ions from the aqueous phase to the surface of the adsorbent material.

#### 3.4.8. CE-Cys Material Reuse Studies

**Figure 12:**
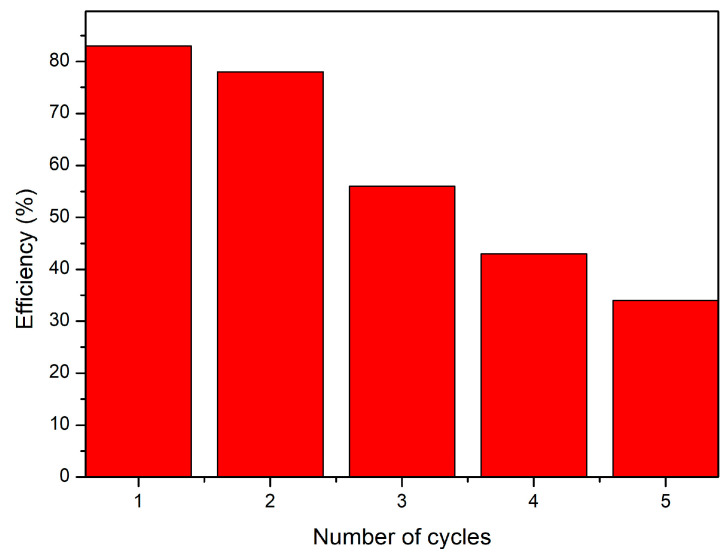
Adsorption–desorption cycles.

The use of material with adsorbent properties in the real adsorption process depends not only on its adsorption capacity but also on its ability to be regenerated and reused. In order to be able to reuse an adsorbent material, it is necessary to be able to easily desorb the Au(III) ions from its surface, in a large quantity, so reuse is profitable. In this sense, the possibility of reusing the CE-Cys material after Au(III) desorption was also investigated, establishing the number of sorption/desorption cycles. The obtained experimental data are presented in Figure 12.

As a result of the studies carried out, the following facts were found:

- With the increase in the number of sorption/desorption cycles, the volume of Au(III) solution passed through the material with adsorbent properties decreases;
- For each adsorption cycle, it was observed that, with the increase in the volume sequence of the added Au(III) solution, the residual concentration of Au(III) ions decreases and the retention efficiency increases until the column breakthrough when the desorption is necessary;
- For each desorption cycle performed, it was observed that the degree of desorption decreases (Figure 12). The maximum number of adsorption/desorption cycles is 5.

## 4. Conclusions

The aim of the present study was to develop new adsorbent materials that can be used for Au(III) ion recovery from aqueous solutions. In order to implement such technologies in current practice, it is necessary that the new produced adsorbent materials are cheap and granular; have a high adsorption capacity, good selectivity, and high physical resistance; and are easy to regenerate for further reuse.

The studies carried out were performed using three different supports and five extractants in different support:extractant ratios. The studied supports were biopolymers— cellulose, chitosan, and diatomaceous—and the used extractants are eco-friendly and relatively cheap amino acids containing pendant groups with nitrogen and sulphur heteroatoms (aspartic acid, L-glutamic acid, valine, DL-cysteine, or serine). All new adsorbent materials were prepared by functionalization, by impregnation, by ultrasonication. Following the preparation, the adsorption test for gold recovery were carried out to select the adsorbent material suitable for Au(III) ion recovery. From all prepared materials, the CE-Cys (in a ratio of support:extractant = 10:1) adsorbent material was found to be the recommended one for Au(III) ion recovery by adsorption.

**Figure 13:**
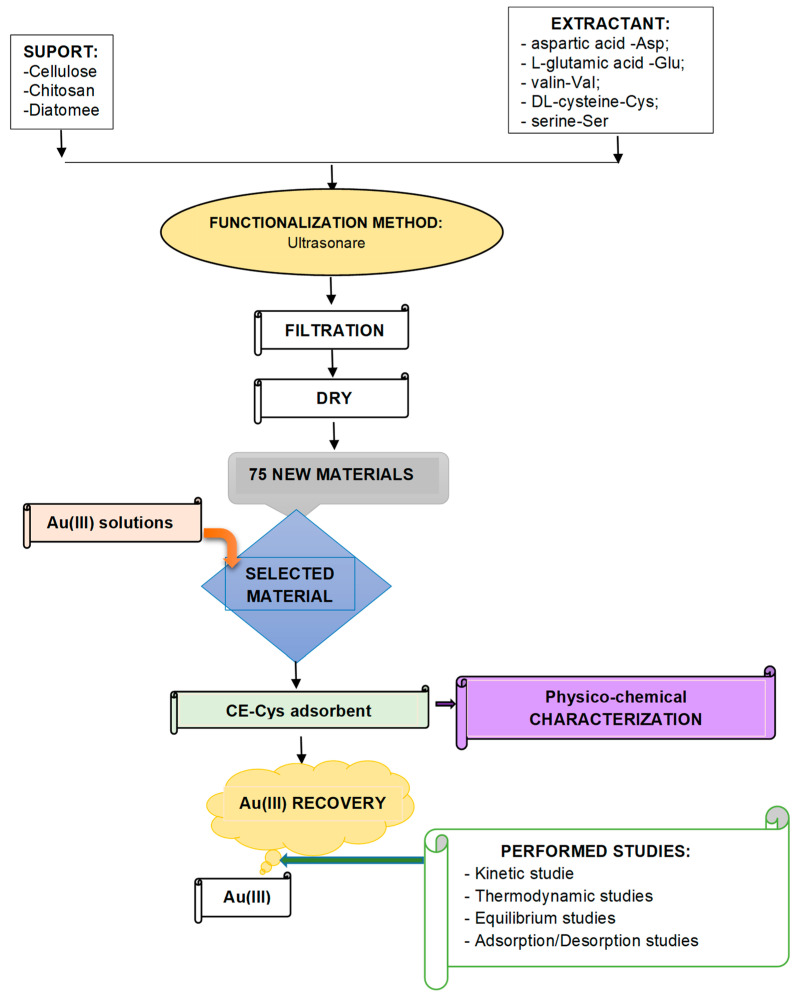
Proposed working flow for design of Au(III) adsorption from aqueous solutions.

Because the aim is represented by the recovery of Au(III) ions from aqueous solutions by adsorption, it must be taken into account that such a process can be influenced by: interactions between different components, various interferences, the presence of different impurities in the adsorbent material, etc. In this context, a working scheme was proposed as a support for a possible design of an adsorptive process (Figure 13).

CE functionalization was confirmed by characterizing the obtained CE-Cys prepared material by physico-chemical methods (SEM—EDX, FTIR). In addition, the zero-charge potential of the surface was determined in order to understand the influence of superficial charge.

Kinetic, thermodynamic, and equilibrium studies were performed in order to establish the Au(III) adsorption mechanism on the newly prepared CE-Cys material. In this context, the influence of the S:L ratio, pH, contact time, temperature, and Au(III) initial concentration on the adsorption capacity were studied. Based on the obtained experimental data, the kinetic parameters associated with the studied process were established, as well as the adsorption isotherm that best describes the adsorption process. Thus, we find out the following:

- The optimal S:L ratio is 0.1 g of adsorbent material:25 mL of gold solution;
- The optimal pH < 4;
- With the increasing contact time, the adsorption capacity increases, reaching equilibrium after 90 min;
- With the temperature increase, an insignificant difference in adsorption capacity was observed;
- The studied adsorption process is accurately shown by the pseudo-second-order kinetic model;
- Since the activation energy calculated for the studied adsorption process is lower than 8 kJ mol^−1^, gold adsorption is a physical process;
- The adsorption data were better modelled by the Sips isotherm;
- The studied material was able to be reused for five adsorption/desorption cycles.

## Figures and Tables

**Table 1:** Kinetic parameters for the adsorption of Au(III) onto CE-Cys adsorbent material.

Pseudo-first-order				
Temperature (K)	*q*_e,exp_ (mg g^−1^)	*k*_1_ (min^−1^)	*q*_e,calc_ (mg g^−1^)	R^2^
298	2.60	0.0093	2.51	0.9055
308	3.01	0.0109	2.73	0.9106
318	3.30	0.0133	3.0	0.9275
328	3.57	0.0167	3.56	0.9771
Pseudo-second-order				
Temperature (K)	*q*_e,exp_ (mg g^−1^)	*k*_2 _ (g mg^−1^·min^−1^)	*q*_e,calc_ (mg g^−1^)	R^2^
298	2.60	0.32	2.01	0.9908
308	3.01	0.72	2.77	0.9905
318	3.30	1.77	3.41	0.9937
328	3.57	3.55	4.33	0.9955
Intraparticle diffusion model (IPD)				
Temperature (K)	K_diff_ (mg·g^−1^ min^−1/2^)		C	R^2^
298	0.030		0.038	0.9057
308	0.050		0.057	0.9000
318	0.064		0.102	0.8663
328	0.079		0.110	0.8864

**Table 2:** Thermodynamic parameters for adsorption of Au(III) onto CE-Cys.

Δ*H*^0^ (kJ/mol)	Δ*S*^0^ (J/mol·K)	Δ*G*^0^ (kJ/mol)				R^2^
24.00	69.6	298 K	308 K	318 K	328K	0.9969
		−20.7	−21.4	−22.1	−22.8	

**Table 3:** Parameters of isotherm model for adsorption of Au(III) onto CE-Cys.

Langmuir isotherm			
q_m,exp_ (mg/g)	K_L_ (L/mg)	q_L_ (mg/g)	R^2^
12.2	0.0311	23.2	0.9715
Freundlich isotherm			
K_F_ (mg/g)	1/n_F_		R^2^
1.26	0.48		0.9693
Sips isotherm			
K_S_	q_S_ (mg/g)	1/n_S_	R^2^
0.03	16.9	0.23	0.9804

## Data Availability

The data presented in this study are available on request from the corresponding author.
